# Acute Kidney Injury and Its Association With Dementia and Specific Dementia Types: Findings From a Population-Based Study in Sweden

**DOI:** 10.1212/WNL.0000000000209751

**Published:** 2024-08-22

**Authors:** Hong Xu, Maria Eriksdotter, Sara Garcia-Ptacek, Daniel Ferreira, Dongze Ji, Annette Bruchfeld, Yang Xu, Juan J. Carrero

**Affiliations:** From the Division of Clinical Geriatrics (H.X., M.E., S.G.-P., D.F.), Department of Neurobiology, Care Sciences and Society, Karolinska Institutet, Solna, Sweden; Department of Pharmacy Administration and Clinical Pharmacy (D.J., Y.X.), School of Pharmaceutical Sciences, Peking University Health Science Center, Beijing, China; Division of Renal Medicine and Baxter Novum (A.B.), Department of Clinical Science, Intervention and Technology, and Department of Medical Epidemiology and Biostatistics (MEB) (J.J.C.), Karolinska Institutet, Solna, Sweden.

## Abstract

**Background and Objectives:**

Preclinical studies suggest that acute kidney injury (AKI) results in biochemical and pathologic changes in the brain. We aimed to explore the association between experiencing AKI and subsequent risks of developing dementia.

**Methods:**

We conducted a study involving individuals aged 65 years and older in Stockholm from 2006 to 2019, who were free from dementia diagnosis and had data on kidney function. The exposure was an episode of AKI (time varying), ascertained by issued clinical diagnoses and transient creatinine elevations according to Kidney Disease Improving Global Outcomes criteria. The outcome was all-cause dementia and specific types of dementia, ascertained by clinically confirmed cases in the Swedish registry of cognitive/dementia disorders, the presence of 2 issued dementia diagnoses in outpatient care, or initiation of specific antidementia medications. We investigated associations with dementia through Cox proportional hazard regression by AKI, severity levels of AKI, AKI recurrence, and setting (community-acquired or hospital-acquired AKI).

**Results:**

We included 305,122 individuals with a median age of 75 ± 8 years (56.6% women). During a median follow-up of 12.3 (interquartile range 8.7–13.3) years, there were 79,888 individuals (26%) suffering from at least 1 episode of AKI and 47,938 incident cases (16%) of dementia. The rate of dementia cases was 37.0 per 1,000 person-years (95% CI 36.2–37.8) after developing AKI, which was approximately 2 times higher than the rate observed during the periods before AKI (17.3, 95% CI 17.2–17.5). After multivariable adjustment, developing AKI was associated with a 49% higher rate of subsequent dementia (adjusted hazard ratio hazard ratio [HR] 1.49, 95% CI 1.45–1.53). This pattern was consistent across dementia types, with HRs of 1.88 (95% CI 1.53–2.32), 1.47 (1.38–1.56), and 1.31 (1.25–1.38) for dementia with Lewy bodies and Parkinson disease with dementia, vascular dementia, and Alzheimer dementia, respectively. Risk associations were stronger in magnitude across more severe AKIs and in hospital-acquired vs community-acquired AKI.

**Discussion:**

Individuals who experienced an AKI were at increased risk of receiving a diagnosis of dementia.

## Introduction

Dementia is characterized by a progressive decline in cognition and functioning that interferes with activities of daily life.^1^ Its prevalence rises with age, affecting between 5% and 50% of individuals aged 65 and older and posing a substantial global health challenge.^2^ The burden of dementia is high, with the number of affected individuals projected to rise from 55 million in 2019 to 150 million in 2050 globally.^3^ Dementia is associated with considerable morbidity and mortality but has limited treatment options,^4^ and therefore, emphasis lies on prevention. So far, various potentially modifiable risk factors have been identified (lower education, hearing loss, traumatic brain injury, midlife hypertension, midlife obesity, alcohol abuse, smoking, depression, social isolation, physical inactivity, air pollution, and diabetes),^4^ which conform the basis for public health prevention campaigns. Ascertaining additional risk factors of dementia is important for identifying high-risk individuals early and advancing preventative and monitoring strategies.

Acute kidney injury (AKI), a sudden (over hours or days) deterioration in kidney function, is relatively common both in-hospital and at the community^5,6^ and is associated with poor in-hospital outcomes and high health care costs.^7^ Individuals who suffer from an AKI continue to be at elevated risk of increased morbidity and mortality,^8^ with impairment at various organs leading to subsequent admission with heart failure,^9^ progression to kidney failure,^10^ and possibly dementia.^11^ Preclinical studies have suggested a potential link between AKI and brain injury, involving mechanisms such as increased vascular permeability, disruption of the blood-brain barrier, peripheral inflammation leading to brain inflammation, hippocampal inflammatory response, retention of neuronal toxins, and reduced central dopamine turnover, resulting in cognitive impairment, anxiety, depression, and subsequent dementia.^12^ Observational studies to date do suggest an association between AKI and future risk of dementia.^13-17^ However, these studies were often affected by low sample sizes, generally used insensitive administrative codes to identify AKI^18^ or dementia,^19^ lacked information on key confounders such as baseline kidney function, and were unable to evaluate the severity of the AKI, the setting (community-acquired vs hospital-acquired), or its recurrence. Furthermore, no clinical studies have explored associations with specific dementia types, and given the detrimental effect of AKI on vascular pathology,^20^ we hypothesized associations, if any, to be stronger with vascular forms of dementia.

Against this background, the objective of this study was to investigate the potential association between AKI, AKI severity, AKI setting, and recurrence, with subsequent risks of developing dementia, encompassing both all-cause dementia incidence and the most common specific dementia types.

## Methods

### Data Source

We used data from the Stockholm CREAtinine Measurement (SCREAM) project, a complete health care utilization cohort of the region of Stockholm, Sweden, capturing interactions with primary, secondary, and tertiary care settings.^21^ A single health care provider in the Stockholm region offers universal and tax-funded health care to 20%–25% of Sweden's population.^21^ Using the unique personal identification number of each citizen, health care encounters and laboratory data were linked to the Swedish registry of cognitive/dementia disorders (SveDem),^22,23^ which includes incident diagnoses of dementia in Sweden, and other regional and national databases containing information on demographics, dispensed drugs, and vital status, with virtually no loss to follow-up.^24^ The regional ethical review board in Stockholm approved the study, and informed patient consent was not required because all data were deidentified after database linkage.

### Study Population

During the study period from January 1, 2006, to December 31, 2018, we included all individuals aged 65 years or older with their first available outpatient creatinine measurement, which was considered the index date and used to estimate the glomerular filtration rate (eGFR).^25^ Next, we excluded individuals with the following conditions: (1) missing information on age or sex; (2) any recorded history of dementia (as identified by issued *International Classification of Diseases, 10th Revision* [*ICD-10*] diagnostic codes, ongoing use of antidementia drugs, or records in SveDem, further detailed in eTable 1); (3) any recorded history of AKI (*ICD-10* code: N17); or (4) undergoing dialysis or had a history of kidney transplantation. To perform these exclusions, we explored medical records before index date and up to 1997, time in which the *ICD-10* version was implemented in Sweden. eFigure 1 illustrates the flowchart of patient selection.

### Standard Protocol Approvals, Registrations, and Patient Consents

The regional ethics committee in Stockholm, Sweden, approved the study protocol. Patient informed consent was not deemed necessary by the ethics committee. Data were deidentified by Swedish government authorities before delivery to the research team.

### Study Exposure

The study exposure was an AKI episode, considered as a time-varying exposure and ascertained by transient creatinine elevations recorded in connection with health care encounters and according to Kidney Disease Improving Global Outcomes (KDIGO) criteria.^26^ The first available outpatient creatinine measurement per patient was considered the “baseline” value. This baseline value determined cohort entry, and it is the time where study covariates were extracted and follow-up began.

During follow-up, we used the algorithm previously developed^27^ to identify AKI events, with minor modifications to suit our data structure (eFigure 2). In this algorithm, each creatinine test during follow-up (named “index” creatinine) is compared in an iterative manner against the “reference” creatinine, which is defined by another outpatient creatinine measurement within a year before each value. When deviations between the index and reference creatinine tests were large enough to meet KDIGO criteria, the AKI event was defined. In some cases, there were no outpatient creatinine tests within a year before, and in that case, the baseline creatinine (at cohort entry) was considered the reference creatinine.

In brief, an AKI event was defined when the index creatinine value met one of the following criteria: (1) ≥1.5 times higher than the reference creatinine, or (2) ≥26.5 μmol/L (0.3 mg/dL) higher than the reference creatinine, and/or (3) initiation of temporary dialysis (*ICD-10* codes T856, T857, Z490, Z992).

The severity stages of the identified AKI episode were determined as follows: stage 1, if the ratio of the index creatinine to the reference creatinine was ≥1.5–2, or if the difference between the index creatinine and the reference creatinine was ≥26.5 μmol/L (0.3 mg/dL); stage 2, if the ratio of the index creatinine to the reference creatinine was ≥2–3; and stage 3, if the ratio of the index creatinine to the reference creatinine was ≥3, or if the index creatinine was 353.6 μmol/L (4 mg/dL), or if temporary dialysis was initiated. AKI episodes were further categorized into different acquired sources: (1) community-acquired/community-managed AKI, where the occurrence and management of AKI were both in a community setting; (2) community-acquired/hospital-managed AKI, where AKI was acquired in the community but with hospital admission within 7 days; and (3) hospital-acquired AKI, where the occurrence of AKI was after 48 hours since hospital admission. In cases where AKI episodes met different severity stages, we selected the most severe stage. If multiple acquired forms were present, we selected the first identified acquired form.

Finally, individuals can experience multiple AKI events, and we hypothesized that AKI recurrence would proportionally correlate with subsequent AKI risk. To test this, we considered the possibility of multiple AKI episodes during follow-up that were categorized as time-varying exposures into 3 ordinal levels: first AKI, second AKI, and ≥third AKI. AKI episodes occurring within a 7-day window were grouped as part of the same event, and the date of the first of these events, in that case, was the event date.

### Study Outcome

The primary study outcome was a clinical diagnosis of dementia, ascertained by registration in the SveDem or the presence of 2 *ICD-10*–based diagnoses of dementia in any source of Swedish health care or the dispensation of antidementia-specific drugs (donepezil, rivastigmine, galantamine, and/or memantine). SveDem captures approximately 75% of primary care and 98% of specialist care facilities of Stockholm involved in the caring of people with dementia, offering extensive but not complete coverage.^28^ Clinical diagnoses and dispensed medications are complete for the region, ascertained with the health care use database of Stockholm's single health care provider, or the Swedish government–run medication register, which records all filled prescriptions in Swedish pharmacies.^29^

The secondary study outcomes included specific dementia types: (1) Alzheimer dementia (AD, including AD and mixed AD dementia that refers to AD plus vascular dementia), (2) vascular dementia (VaD), (3) dementia with Lewy bodies (DLB) and Parkinson disease with dementia (PDD), (4) frontotemporal dementia (FTD), (5) unspecified dementia, and (6) other types such as corticobasal syndrome or alcohol-related dementia. The etiology of dementia was primarily obtained from diagnostic workups and subsequent specified dementia diagnoses in SveDem. While SveDem aims to capture all dementia diagnoses in Sweden, political and organizational challenges hinder this goal. Therefore, we also considered the inclusion of additional dementia events captured by the Swedish government–run national patient registry of outpatient-specialist and hospital diagnoses (through *ICD-10* codes) and the initiation of dementia-specific medications captured by the government-run prescribed medication registry (through Anatomical Therapeutic Chemical codes). We did neither consider “unspecified dementia” cases (a term used to classify dementia cases that do not fit well-defined subtypes such as AD or VaD^1^), nor other rarer forms of dementia such as alcohol-related dementia.

Follow-up started at the index date and ended at the end of the follow-up period (December 31, 2019), death, or migration from the region, whichever occurred first. The date of death was obtained from the National Board of Health and Welfare's Cause-of-Death Register.^30^

### Covariates

Covariates were defined at baseline and time-updated at the time of an AKI episode. Covariates included age, sex, baseline or reference eGFR (with the first available outpatient creatinine measurement was considered the baseline eGFR and the outpatient eGFR within a year before the AKI was considered the reference eGFR), selected comorbidities (tobacco abuse, alcohol abuse, obesity, hypertension, diabetes, congestive heart failure, myocardial infarction, stroke, atrial fibrillation, cancer, depression, hearing loss, and traumatic brain injury), and medications (angiotensin-converting enzyme inhibitor/angiotensin receptor blocker [ACEi/ARB], β-blocking agents, calcium channel blockers, nonsteroidal anti-inflammatory drugs [NSAIDs], and statins). Definitions of comorbidities and medications are detailed in eTable 2.

### Statistical Analysis

Values were expressed as mean and SD for continuous variables with a normal distribution, median (interquartile range, IQR) for non-normal distribution variables, and percentage of the total for categorical variables at baseline and the time of AKI episode.

The AKI episode was treated as a time-varying variable in Cox proportional hazard regression models, with attained (chronological) age as the underlying timescale to evaluate the association between AKI and dementia. Using age as the timescale, we inherently adjust for age at entry. Next, we used the same strategy to investigate associations of AKI of differing severity (AKI stage 1 and AKI stages 2 and 3 are combined to increase statistical efficiency and power) and different acquired sources (community-acquired/community-managed, community-acquired/hospital-managed, and hospital-acquired). Finally, we implemented a cumulative exposure metric to track AKI episodes, creating a longitudinal, continuous variable that captured the most recent cumulative AKI occurrences until a given time t (referred to as cumulative AKI episodes). The models adjusted for all covariates described in the “Covariates” section. Because a low kidney function is an important predictor of AKI risks,^31^ we adjusted for both baseline eGFR at cohort entry and reference eGFR at the time of AKI development.

### Subgroup Analyses

We explored the consistency of our findings across the following strata: (1) age at index date (65–74, 75–84, ≥85 years); (2) sex (female or male); (3) baseline kidney function (eGFR strata of >90, 60–89, 45–59, 30–44, and <30 mL/min/1.73 m^2^ as per KDIGO criteria)^32^; and presence/absence of clinical diagnoses of (4) hypertension, (5) diabetes, (6) stroke, (7) depression, (8) traumatic brain injury, and (9) hearing loss. We tested for the presence of multiplicative interactions between AKI and these strata in their association with dementia.

### Sensitivity Analyses

We then performed a series of sensitivity analyses to test the robustness of our results. First, we excluded dementia cases that occurred within 90 days from the first AKI date to address reverse causation bias (i.e., that the AKI was detected during the examinations toward a diagnosis of dementia). Second, to address the potential bias caused by informative censoring, we reanalyzed our data using inverse probability censoring weighting (eMethods).^33,34^ The rationale is that death is a likely outcome of both AKI and dementia and common in older adults, possibly inducing this informative censoring.

Third, we calculated the E-values for the outcome of all-cause dementia. The E-value is a statistical measure to assess the robustness of observed associations to potential unmeasured confounders. The E-value quantifies the minimum strength of association of an unmeasured confounder with the exposure (AKI) and outcome (dementia) needed to explain the observed association. Higher E-values indicate greater robustness of the association. However, the E-value may represent the effect of 1 confounder or the composite effect of multiple unmeasured confounders. Finally, to assess the potential impact of unmeasured factors related to AKI, we used psoriasis and the need for cataract surgery as negative control outcomes. Because the etiology and pathophysiology of AKI are unrelated to psoriasis or cataract surgery, we did not expect these conditions to be associated. In addition, hospitalization due to heart failure served as a positive control outcome, given the shared pathophysiologic mechanisms and the well-established link between these 2 conditions.^35^ Any presence of a significant association (or lack of it) in these scenarios might suggest remaining confounding.^36^

All study covariates were complete to the extent that were recognized and coded in health care; there were no missing values. All analyses were performed using R 3.4.3 software (The R Project for Statistical Computing, Vienna, Austria).

### Data Availability

SCREAM study data are stored at the Department of Medical Epidemiology and Biostatistics at Karolinska Institutet (ki.se/meb) and can be made available to academic researchers for collaborative projects on request.

## Results

### Characteristics of Individuals at Study Inclusion and Time of AKI Episode

After applying inclusion and exclusion criteria, a total of 305,122 older adults with at least 1 outpatient creatinine measurement to estimate kidney function were included in our analysis. Their mean age was 75 ± 8 years, and 56.6% were women. Baseline characteristics are provided in Table 1. The most common comorbidity was hypertension (35.9%), followed by diabetes (11.1%) and atrial fibrillation (10.1%). The use of β-blockers and angiotensin-converting enzyme inhibitors and angiotensin II receptor blockers (ACEi and ARB) was frequent, accounting for 29.7% and 25.0% of individuals, respectively. In total, 19.7% of individuals had CKD stages 3–5.

**Table 1:** Characteristics of Individuals at Study Inclusion and at the Time of AKI Episode

Characteristics	At study inclusion (n = 305,122)	At time of AKI occurrence (n = 79,888)
Demographics		
Age, y, mean (SD)	75 (8)	77 (8)
Age categories, n (%)		
65–74 y	164,845 (54.0)	33,264 (41.6)
75–84 y	97,272 (31.9)	31,850 (39.9)
≥85 y	43,005 (14.1)	14,774 (18.5)
Female, n (%)	172,598 (56.6)	42,499 (53.2)
Comorbidities, n (%)		
Tobacco abuse	1,255 (0.4)	1,589 (2.0)
Alcohol abuse	6,383 (2.1)	3,958 (5.0)
Obesity	5,887 (1.9)	4,299 (5.4)
Hypertension	109,597 (35.9)	55,338 (69.3)
Diabetes mellitus	33,897 (11.1)	20,334 (25.5)
Congestive heart failure	26,954 (8.8)	27,137 (34.0)
Myocardial infarction	22,465 (7.4)	16,117 (20.2)
Stroke	18,492 (6.1)	12,691 (15.9)
Atrial fibrillation	30,750 (10.1)	23,889 (29.9)
Cancer	28,220 (9.2)	20,605 (25.8)
Depression	14,776 (4.8)	9,864 (12.3)
Hearing loss	23,399 (7.7)	13,599 (17.0)
Traumatic brain injury	16,035 (5.3)	10,699 (13.4)
Concurrent medication use, n (%)		
ACEi and ARB	76,390 (25.0)	42,877 (53.7)
β-blocker	90,498 (29.7)	41,555 (52.0)
CCB	47,366 (15.5)	23,132 (29.0)
NSAID	63,535 (20.8)	16,115 (20.2)
Statin	9,157 (3.0)	5,526 (6.9)
Kidney function tests		
eGFR, mL/min/1.73 m^2^, median (IQR)	77.3 (63.8, 87.3)	49.1 (33.6, 70.5)
CKD stage, n (%)^a^		
G1	52,107 (17.1)	4,615 (5.8)
G2	192,988 (63.2)	24,082 (30.1)
G3a	39,881 (13.1)	16,198 (20.3)
G3b	15,183 (5.0)	19,489 (24.4)
G4–5	4,963 (1.6)	15,504 (19.4)

Abbreviations: ACEi = angiotensin-converting enzyme inhibitor; AKI = acute kidney injury; ARB = angiotensin II receptor blocker; CCB = calcium channel blocker; CKD = chronic kidney disease; eGFR = estimated glomerular filtration rate; NSAID = nonsteroidal anti-inflammatory drug.

a *p* < 0.001

During a median follow-up of 12.3 (IQR 8.7–13.3) years, 79,888 individuals (26.2%) experienced at least 1 episode of AKI, with an overall AKI incidence of 24.71 per 1,000 person-years (95% CI 24.54–24.88). At the time of AKI occurrence, the mean age was 77 ± 8 years, with a lower proportion of women (53.2%) compared with the individuals who did not experience any AKI. Their most common comorbidity was hypertension but with a higher prevalence (69.3%) than individuals at study inclusion. ACEs and ARBs (53.7%) and β-blockers (52%) were also the first 2 frequently used drugs. The proportion of individuals with CKD stages 3–5 was higher (64.1%).

### Evaluation of Data Set Representativeness

During the period of data collection, there were 324,013 individuals aged 65 years or older who accessed health care in the region of Stockholm. Of those, 94% had at least 1 creatinine measurement taken and entered in our study. Their demographics are similar to the 6% of individuals not included in our study due to lack of creatinine tested (eTable 3).

### Association Between AKI and All-Cause Dementia

Throughout the follow-up period, a total of 47,938 all-cause dementia events (15.7%) were recorded. The incidence rate was 2-fold higher after an AKI episode (36.99 per 1,000 person-years, 95% CI 36.23–37.75) compared with before (17.34 per 1,000 person-years, 95% CI 17.17–17.52). After adjusting for age, sex, reference eGFR, selected comorbidities, and medications (all those listed under the section “Covariates”), suffering from AKI was associated with a subsequent 1.5-fold higher rate of all-cause dementia (hazard ratio [HR] 1.49, 95% CI 1.45–1.53) (Table 2).

**Table 2:** Association Between Developing AKI and Risk of All-Cause Dementia

Periods	No. of individuals	No. of dementia events	Follow-up time, y, median (IQR)	Incidence rate/1,000 PY and 95% CI	Adjusted HR^a^ (95% CI)
Non-AKI periods	305,122	38,836	7.43 (3.13–11.81)	17.34 (17.17–17.52)	Ref
All AKI episodes					
After AKI	79,888	9,102	1.92 (0.26–4.93)	36.99 (36.23–37.75)	1.49 (1.45–1.53)
AKI severity					
AKI stage 1	58,139	6,963	2.17 (0.41–5.20)	36.62 (35.77–37.49)	1.45 (1.41–1.50)
AKI stages 2 and 3	21,749	2,139	1.18 (0.07–4.15)	38.21 (36.63–39.85)	1.61 (1.53–1.68)^b^
AKI acquired form					
Community-acquired and community-managed	21,411	2,579	2.92 (0.94–6.21)	30.67 (29.51–31.86)	1.31 (1.26–1.37)
Community-acquired and hospital-managed	37,276	4,305	1.72 (0.19–4.57)	40.19 (39.01–41.4)	1.56 (1.51–1.62)^c^
Hospital-acquired	21,201	2,218	1.26 (0.11–4.12)	40.41 (38.76–42.11)	1.54 (1.47–1.61)^c^
AKI recurrence					
1 AKI event	79,888	6,129	0.70 (0.06–3.12)	36.51 (35.61–37.43)	1.47 (1.43–1.52)
2 AKI events	34,724	1,692	0.22 (0.04–1.53)	39.79 (37.94–41.70)	1.48 (1.41–1.56)
≥3 AKI events	60,332	1,281	0.09 (0.03–0.41)	35.90 (33.99–37.89)	1.39 (1.31–1.48)

Abbreviations: ACEi = angiotensin-converting enzyme inhibitor; AKI = acute kidney injury; ARB = angiotensin II receptor blocker; CI = confidence interval; eGFR = estimated glomerular filtration rate; HR = hazard ratio; IQR = interquartile range; NSAID = nonsteroidal anti-inflammatory drug; PY = person-years.

a Adjusted for age, sex, baseline or reference eGFR (the first available outpatient creatinine measurement was considered the baseline eGFR and eGFR within a year before the AKI was considered the reference eGFR), selected comorbidities (tobacco abuse, alcohol abuse, obesity, hypertension, diabetes, congestive heart failure, myocardial infarction, stoke, atrial fibrillation, cancer, depression, hearing loss, and traumatic brain injury), and medications (ACEi/ARB, β-blocking agents, calcium channel blockers, NSAIDs, and statins).

b Statistically different (*p* < 0.05) from AKI stage 1.

c Statistically different (*p* < 0.05) from community-acquired/community-managed AKI.

There was a suggestion of a dose-response effect, with higher dementia risk magnitudes across more severe AKIs from a 1.45-fold (95% CI 1.41–1.50) higher risk after AKI stage 1, to a 1.61-fold (95% CI 1.53–1.68) higher risk after AKI stages 2 and 3 (noncrossing confidence intervals indicate statistical difference from one another; Table 2). In addition, we also observed a higher risk after community-acquired/hospital-managed AKI (HR 1.56, 95% CI 1.51–1.62) and hospital-acquired AKI (HR 1.54, 95% CI 1.47–1.61) compared with community-acquired/community-managed AKI (HR 1.31, 95% CI 1.26–1.37). The risk after recurrent AKI episodes was, however, similarly elevated: individuals with 1 AKI reported 47% higher risk of all-cause dementia (HR 1.47, 95% CI 1.43–1.52), individuals with 2 recurrent AKIs had 48% higher risk (HR 1.48, 95% CI 1.41–1.56), and individuals with 3 or more recurrent AKIs had 39% higher risk (HR 1.39, 95% CI 1.31–1.48) (Table 2).

### Association Between AKI and Specific Dementia Types

Among dementia events, 26,879 had a specific type diagnosed. AD was the most common specific dementia type reported (n = 17,663, accounting for 65.7% of specific dementia types), followed by VaD (n = 7,971, 29.7% of specific dementia types), DLB and PDD (n = 975, 3.6% of specific dementia types), and FTD (n = 270, 1% of specific dementia types). The incidence rates of all specific dementia types increased after AKI. The risk magnitudes were highest for DLB and PDD (HR 1.88, 95% CI 1.53–2.32), followed by VaD (HR 1.47, 95% CI 1.38–1.56) and AD (HR 1.31, 95% CI 1.25–1.38). We did not find a statistically significant association between AKI and FTD (HR 1.44, 95% CI 0.92–2.25), but risk magnitudes were also elevated (Table 3).

**Table 3:** Association Between AKI Episode and Risk of Specific Type of Dementia

Periods	No. of individuals	No. of dementia events	Follow-up time, y, median (IQR)	Incidence rate/1,000 PY and 95% CI	Adjusted HR^a^ (95% CI)
AD					
Non-AKI periods	305,122	15,235	7.43 (3.13–11.81)	6.8 (6.7–6.91)	Ref
AKI periods	79,888	2,428	1.92 (0.26–4.93)	9.87 (9.48–10.26)	1.31 (1.25–1.38)
VaD					
Non-AKI periods	305,122	6,118	7.43 (3.13–11.81)	2.73 (2.66–2.8)	Ref
AKI periods	79,888	1,853	1.92 (0.26–4.93)	7.53 (7.19–7.88)	1.47 (1.38–1.56)
DLB and PDD					
Non-AKI periods	305,122	831	7.43 (3.13–11.81)	0.37 (0.35–0.4)	Ref
AKI periods	79,888	144	1.92 (0.26–4.93)	0.59 (0.5–0.68)	1.88 (1.53–2.32)
FTD					
Non-AKI periods	305,122	241	7.43 (3.13–11.81)	0.11 (0.09–0.12)	Ref
AKI periods	79,888	29	1.92 (0.26–4.93)	0.12 (0.08–0.16)	1.44 (0.92–2.25)

Abbreviations: ACEi = angiotensin-converting enzyme inhibitor; AD = Alzheimer disease; AKI = acute kidney injury; ARB = angiotensin II receptor blocker; DLB = dementia with Lewy bodies; eGFR = estimated glomerular filtration rate; FTD = frontotemporal dementia; HR = hazard ratio; IQR = interquartile range; NSAID = nonsteroidal anti-inflammatory drug; PDD = Parkinson disease dementia; PY = person-years; VaD = vascular dementia.

a Adjusted for age, sex, baseline or reference eGFR (the first available outpatient creatinine measurement was considered the baseline eGFR and eGFR within a year before the AKI was considered the reference eGFR), selected comorbidities (tobacco abuse, alcohol abuse, obesity, hypertension, diabetes, congestive heart failure, myocardial infarction, stoke, atrial fibrillation, cancer, depression, hearing loss, and traumatic brain injury), and medications (ACEi/ARB, β-blocking agents, calcium channel blockers, NSAIDs, and statins).

Likewise, we generally observed higher risk of specific dementia types after more severe AKI episodes and after AKI episodes requiring hospitalization, although CIs broadened with lower power by stratification. For recurrent AKI events, risk associations were similarly elevated and not different across more recurrent events (eTable 4).

### Subgroup and Sensitivity Analyses

**Figure 1:**
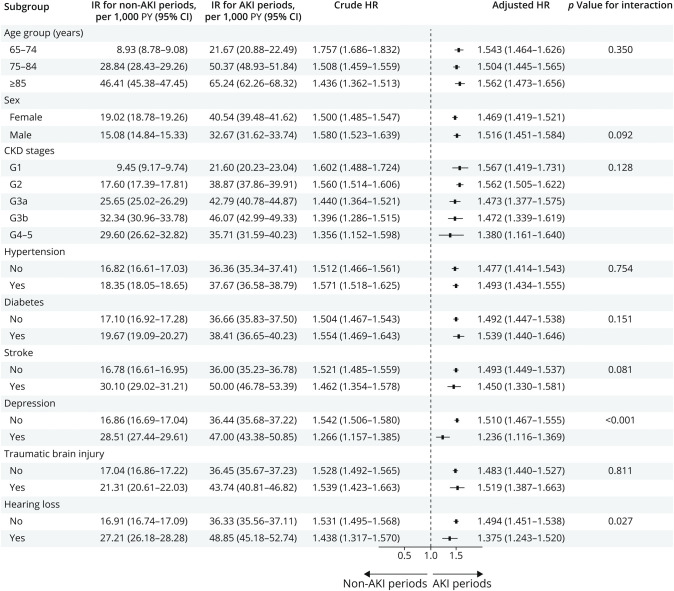
Subgroup Analyses Regarding the Association Between Developing AKI and Risk of All-Cause Dementia ACEi = angiotensin-converting enzyme inhibitor; AKI = acute kidney injury; ARB = angiotensin II receptor blocker; eGFR = estimated glomerular filtration rate; HR = hazard ratio; NSAID = nonsteroidal anti-inflammatory drug; PY = person-years. ^†^Adjusted for age, sex, baseline or reference eGFR (the first available outpatient creatinine measurement was considered the baseline eGFR and eGFR within a year before the AKI was considered the reference eGFR), selected comorbidities (tobacco abuse, alcohol abuse, obesity, hypertension, diabetes, congestive heart failure, myocardial infarction, stoke, atrial fibrillation, cancer, depression, hearing loss, and traumatic brain injury), and medications (ACEi/ARB, β-blocking agents, calcium channel blockers, NSAIDs, and statins).

Subgroup analyses (Figure 1) suggested consistency in our observations across strata of age (65–74, 75–84, or ≥85 years); sex (female or male); baseline kidney function; and history of hypertension, diabetes, stroke, and traumatic brain injury. The association between AKI and dementia seemed to be stronger in people without depression and hearing loss compared with people with these conditions (*p* for interaction <0.05). However, in both strata (with and without the condition), the hazard ratios were more than one suggesting consistently higher risks. This pattern was different among specific types of dementia (eTable 5): we observed that the risk associated with dementia with Lewy bodies and Parkinson disease was higher in individuals older than 85 years (HR 4.15, 95% CI 1.81–9.54).

Results were consistent after excluding early dementia events occurring within 90 days from the AKI event (HR 1.21, 95% CI 1.17–1.24), suggesting that reverse causation bias may not be high (eTable 6). Throughout the follow-up period, 111,096 individuals (44.7%) died. AKI was associated with 18% higher risk of all-cause dementia (HR 1.18, 95% CI 1.16–1.21) after accounting for death as a competing risk. The effect size decreased by 26% compared with that not accounting for death (eTable 7). The E-value of AKI for the risk of all-cause dementia was 2.33, with point estimates at 2.25 at the lower confidence limits. By comparing the E-value with the effect size of the other confounders in the multivariable-adjusted Cox regression model, we interpreted the risk of unmeasured confounding to be possibly low (eTable 8). Experiencing AKI was not associated with the risk of being diagnosed with psoriasis (HR 0.94, 95% CI 0.87–1.02) or cataract (HR 1.01, 95% CI 0.99–1.03), but it was associated with the risk of heart failure (HR 4.10, 95% CI 4.02–4.18) (eTable 9).

## Discussion

In this large region-representative study, individuals who experienced an episode of AKI were at an increased risk of receiving a diagnosis of dementia during follow-up. This association was stronger across more severe stages of AKI, through AKIs requiring hospitalization (vs those managed in outpatient care), and across specific dementia types. This study thus identifies individuals with AKI as a population at high risk of dementia who may benefit from close monitoring for early detection and implementation of antidementia strategies.

Our study has both confirmatory and novel findings. We confirm an association between AKI and the risk of dementia.^13-17^ Previous studies identified AKI throughout inpatient clinical diagnoses and matched them with controls. For example, a study from the National Health Insurance database of Taiwan and involving 400,000 adults reported a nearly 2-fold increased risk of dementia in individuals with hospitalized AKI identified using ICD codes, compared with propensity score–matched individuals without AKI, during a 12-year follow-up period.^13^ Instead of matching analysis, we adopted a time-varying design to accommodate changes in AKIs over time and better capture the risk of the nonexposed population. A report from the Atherosclerosis Risk in Communities (ARIC) study, a US screening cohort of 11,074 individuals (mean age 63 years, 56% female), used a similar time-varying design as ours and observed 69% higher dementia risk after an inpatient AKI diagnosis.^14^ Identified strengths of our study include its large sample size and long follow-up period, as well as evaluation of AKI through laboratory tests. We also see as a strength the unique setting involving real-world individuals from a country with universal tax-funded health care, which minimizes selection bias from disparate access to health care.

Our study presents some novel findings, involving an observed “dose-response” effect of AKI on dementia risk, with higher relative risk magnitudes across more severe AKIs (compared with less severe AKIs) or across AKIs requiring hospitalization (compared with those managed in outpatient settings). “Dose responses” add credible support to the hypothesis that these associations may be, at least in part, causal. Previous studies were unable to ascertain AKI severity throughout ICD codes and focused on hospital-acquired AKI.^13-17^ Furthermore, ICD codes are insensitive^18^ and fail to capture many AKI events, particularly those occurring in the community. We reflect that our findings have some similarity with 2 preceding reports observing associations between AKI stages 2 and 3 and the risk of delirium, but no association was found for AKI stage 1.^37,38^ The risk of dementia after recurrent AKIs was also elevated, but we did not observe major differences in magnitude after having 2, 3, or more recurrent AKIs. We recognize that low power and survivor biases may affect our results on AKI recurrence, and these should be interpreted cautiously.

Another novel finding is the observation that AKI was associated with a variety of specific dementia types. We initially hypothesized associations between AKI and dementia to be stronger for vascular dementias, due to the similarities—and potential synergisms—between the vascular pathophysiologic mechanisms underlying both conditions.^39^ However, besides the hypothesized association with vascular dementia, we also observe associations between AKI and DLB and PDD. Underlying mechanisms may not be clear, but we note that a significant number of individuals with DLB and PDD suffer from coexistent cerebrovascular pathology and this phenomenon becomes more pronounced with increasing age.^1,40-43^ In our stratified analyses (eTable 4), we did observe an excess risk associated with DLB and PDD in those older than 85 years compared with younger age strata. Second, postganglionic sympathetic cardiac denervation and severe autonomic dysfunction (including orthostatic hypotension) are important features of DLB and PDD,^44^ which are also common features after AKI.^45^ In addition, in preclinical studies, rat models of bilateral ischemia-reperfusion injury-induced AKI exhibited impaired locomotor activity, reduced central dopamine turnover, and degeneration of dopaminergic neurons,^46^ which are core features of DLB and PDD. Vascular brain injury is central to VaD, but Lewy body pathologic features are not significant in individuals with VaD. Conversely, vascular brain injury has a smaller impact on individuals with AD, which may explain the observed decrease in risk in our study from DLB to VaD and the subsequent decrease to AD.^47^ The lack of statistical significance in the association between AKI and FTD may be in part related to low power. Collectively, our analysis of specific dementia subtypes provides potential mechanism insight into the link between AKI and dementia, possibly involving vascular complications, lack of neurotransmitters, neuroinflammation, and autonomic imbalance.

The strength of our study lies in its large sample size and long follow-up. Our study is not exempt from limitations. First, the links established in our study regarding AKI and dementia may not be causal. Second, the date of the dementia diagnosis may not reflect the date when neurodegenerative disease (NDD), such as Alzheimer disease or Parkinson disease, started, and people suffering from NDD may not be recognized in health care with a clinical diagnosis (i.e., outcome misclassification bias). Third, clinically diagnosed alcoholism, smoking, and obesity ascertained through *ICD-10* codes may be specific, but not sensitive, and we lacked information on lifestyle habits; people living in institutions; cognitive data at baseline; and potential mediators and modifiers such as urinary tract infections, blood lipids, APOE genotype, or homocysteine concentration,^48^ all of which could affect our findings and should be evaluated in future studies. In addition, adjusting for the timing of eGFR measurements relative to the AKI episode (reference eGFR) can introduce model complexities. Fourth, an important limitation of our study is the inherent challenges of accuracy in dementia diagnoses. While SveDem diagnoses are based on routine clinical practice and are believed to be relatively accurate, ICD codes, especially non-SveDem diagnoses for specific dementia types, may not always be accurate. Therefore, results regarding specific dementia subtypes should be interpreted with caution. Finally, our results may represent Stockholm health care during a defined period. Extrapolation to other regions and countries of ethnic diversity should be performed with caution.

In conclusion, individuals who experienced AKI were at increased risk of receiving a clinical diagnosis of dementia. This association was present for the most common dementia types and was stronger across more severe AKIs and AKI requiring hospitalization or managed in hospital. As a clinical application, this study thus identifies individuals with AKI as a population in which monitoring for dementia and potential preventive and therapeutic strategies may be indicated.

## Appendix Authors

**Table:**

Name	Location	Contribution
Hong Xu, MD, PhD	Division of Clinical Geriatrics, Department of Neurobiology, Care Sciences and Society, Karolinska Institutet, Solna, Sweden	Drafting/revision of the manuscript for content, including medical writing for content; major role in the acquisition of data; study concept or design; analysis or interpretation of data
Maria Eriksdotter, MD, PhD	Division of Clinical Geriatrics, Department of Neurobiology, Care Sciences and Society, Karolinska Institutet, Solna, Sweden	Drafting/revision of the manuscript for content, including medical writing for content; major role in the acquisition of data
Sara Garcia-Ptacek, MD, PhD	Division of Clinical Geriatrics, Department of Neurobiology, Care Sciences and Society, Karolinska Institutet, Solna, Sweden	Drafting/revision of the manuscript for content, including medical writing for content; major role in the acquisition of data
Daniel Ferreira, PhD	Division of Clinical Geriatrics, Department of Neurobiology, Care Sciences and Society, Karolinska Institutet, Solna, Sweden	Drafting/revision of the manuscript for content, including medical writing for content
Dongze Ji, MPH	Department of Pharmacy Administration and Clinical Pharmacy, School of Pharmaceutical Sciences, Peking University Health Science Center, Beijing, China	Analysis or interpretation of data
Annette Bruchfeld, MD, PhD	Division of Renal Medicine and Baxter Novum, Department of Clinical Science, Intervention and Technology, Karolinska Institutet, Solna, Sweden	Drafting/revision of the manuscript for content, including medical writing for content
Yang Xu, PhD	Department of Pharmacy Administration and Clinical Pharmacy, School of Pharmaceutical Sciences, Peking University Health Science Center, Beijing, China	Drafting/revision of the manuscript for content, including medical writing for content; study concept or design; analysis or interpretation of data
Juan J. Carrero, Pharm PhD	Department of Medical Epidemiology and Biostatistics (MEB), Karolinska Institutet, Solna, Sweden	Drafting/revision of the manuscript for content, including medical writing for content; major role in the acquisition of data

## Glossary

ACEi: angiotensin-converting enzyme inhibitor
AD: Alzheimer dementia
AKI: acute kidney injury
ARB: angiotensin receptor blocker
DLB: dementia with Lewy bodies
eGFR: estimated glomerular filtration rate
FTD: frontotemporal dementia
HR: hazard ratio
*ICD-10*: *International Classification of Diseases, 10th Revision*
IQR: interquartile range
KDIGO: Kidney Disease Improving Global Outcomes
NDD: neurodegenerative disease
NSAID: nonsteroidal anti-inflammatory drug
PDD: Parkinson disease with dementia
SCREAM: Stockholm CREAtinine Measurement
SveDem: Swedish registry of cognitive/dementia disorders
VaD: vascular dementia
